# Micro-CT Based Experimental Liver Imaging Using a Nanoparticulate Contrast Agent: A Longitudinal Study in Mice

**DOI:** 10.1371/journal.pone.0025692

**Published:** 2011-09-30

**Authors:** Hanne Boll, Stefanie Nittka, Fabian Doyon, Michael Neumaier, Alexander Marx, Martin Kramer, Christoph Groden, Marc A. Brockmann

**Affiliations:** 1 Department of Neuroradiology, Medical Faculty Mannheim, University of Heidelberg, Mannheim, Germany; 2 Department of Clinical Chemistry, Medical Faculty Mannheim, University of Heidelberg, Mannheim, Germany; 3 Department of Surgery, Medical Faculty Mannheim, University of Heidelberg, Mannheim, Germany; 4 Department of Pathology, Medical Faculty Mannheim, University of Heidelberg, Mannheim, Germany; 5 Small Animal Clinic, Department of Veterinary Clinical Sciences, Justus-Liebig-University, Giessen, Germany; University of Helsinki, Finland

## Abstract

**Background:**

Micro-CT imaging of liver disease in mice relies on high soft tissue contrast to detect small lesions like liver metastases. Purpose of this study was to characterize the localization and time course of contrast enhancement of a nanoparticular alkaline earth metal-based contrast agent (VISCOVER ExiTron nano) developed for small animal liver CT imaging.

**Methodology:**

ExiTron nano 6000 and ExiTron nano 12000, formulated for liver/spleen imaging and angiography, respectively, were intravenously injected in C57BL/6J-mice. The distribution and time course of contrast enhancement were analysed by repeated micro-CT up to 6 months. Finally, mice developing liver metastases after intrasplenic injection of colon carcinoma cells underwent longitudinal micro-CT imaging after a single injection of ExiTron nano.

**Principal Findings:**

After a single injection of ExiTron nano the contrast of liver and spleen peaked after 4–8 hours, lasted up to several months and was tolerated well by all mice. In addition, strong contrast enhancement of abdominal and mediastinal lymph nodes and the adrenal glands was observed. Within the first two hours after injection, particularly ExiTron nano 12000 provided pronounced contrast for imaging of vascular structures. ExiTron nano facilitated detection of liver metastases and provided sufficient contrast for longitudinal observation of tumor development over weeks.

**Conclusions:**

The nanoparticulate contrast agents ExiTron nano 6000 and 12000 provide strong contrast of the liver, spleen, lymph nodes and adrenal glands up to weeks, hereby allowing longitudinal monitoring of pathological processes of these organs in small animals, with ExiTron nano 12000 being particularly optimized for angiography due to its very high initial vessel contrast.

## Introduction

Non-invasive longitudinal monitoring of pathological processes in small animal models using micro-computed tomography (micro-CT) has gained increasing importance within the last decade [1]. For micro-CT imaging of liver disease in small animals, high soft tissue contrast is required in order to detect small pathological lesions such as liver metastases. For this purpose, contrast agents providing a positive contrast of the liver have been developed for use in micro-CT in small animals [2], [3], [4], [5], [6], [7], [8], [9], [10], [11], [12], [13], [14]. Since the hitherto used contrast agents have relatively short elimination times on the order of hours and up to a few days, they generally have to be injected time and again prior to each examination [2], [4], [14], [15]. As repeated tail vein injections in mice are time-consuming, a burden for the animal and still include the risk of false injection, a longer-lasting contrast of the liver would be preferable for fast, easy, and repetitive micro-CT studies in small animal models of liver disease.

In the present study the time-course and distribution of contrast enhancement after a single intravenous (i.v.) injection of a nanoparticular contrast agent in mice is characterized. Furthermore, the use of the contrast agent for the detection of liver metastases in mice using micro-CT is demonstrated.

## Materials and Methods

### Alkaline earth metal-based nanoparticulate contrast agent

The contrast agent used in the present study (Viscover™ ExiTron™ nano; Miltenyi Biotec, Bergisch-Gladbach, Germany) is an alkaline earth-based nanoparticulate contrast agent specifically formulated for pre-clinical computed tomography imaging. The nanoparticles are sterically stabilized by a polymer coating and have a mean hydrodynamic diameter of 110 nm. Upon i.v. injection, ExiTron nano circulates in the blood stream and is taken up by cells of the reticuloendothelial system (RES), including macrophages within the liver, the so-called Kupffer cells. Two different formulations of the contrast agent are available and both were used in the underlying study: ExiTron nano 6000 (optimized for liver/spleen imaging) and ExiTron nano 12000 (optimized for angiography) with densities of the undiluted contrast agents prior to injection of approx. 6000 HU and approx. 12000 HU, respectively. The injected volume of 100 µl ExiTron nano per mouse (25g) corresponds to a dose equivalent to 640 mg iodine/kg body weight or 1200 mg iodine/kg body weight for ExiTron nano 6000 and ExiTron nano 12000, respectively.

### Anaesthesia, intubation, and micro-CT imaging of mice

All experiments were carried out after receiving the local ethics committee approval (Regierungspräsidium Karlsruhe; G-202/10). Institutional guidelines for animal welfare and experimental conduct were followed. The animals were anesthetized by isoflurane (Forene; Servopharma GmbH, Oberhausen, Germany) inhalation (3% for induction and 1–2% for maintenance) and transferred to a prewarmed animal operation warmth plate (MEDAX GmbH, Neumuenster, Germany). A catheter for i.v. injection of the contrast agent was inserted as previously described [16]. Briefly, a mouse-tourniquet was tightened over the base of the tail and a 27-gauge i.v. catheter (pre-flushed with a heparin solution) was inserted into a lateral tail vein and fixed with tape.

To reduce artefacts arising from respiratory motion, for some of the CT scans the mice were intubated and ventilated as described previously [17], [18]. Briefly, for intubation, mice were placed supine on a tilted heating plate. A strong light source allowed transillumination of the trachea. The tongue was gently pulled out in order to reveal a view on the vocal cords. A shortened neuroradiological wire guide with an extremely soft tip (Mirage 0.008 in.; Micro Therapeutics, Grenoble Cedex, France) was used for atraumatic intubation. A 22-G i.v. catheter (Klinika Medical GmbH, Germany) was modified by attaching a silicone wedge as described by MacDonald et al. [19] and pushed over the guide wire using Seldingeŕs technique. The endotracheal tube was connected to a small animal ventilator (Small Animal Ventilator KTR5; Hugo Sachs Elektronik-Harvard Apparatus, March-Hugstetten, Germany) and mice were ventilated at a respiratory rate of 100 breaths per minute with a ventilatory tidal volume of 0.2 ml. Relaxation by i.p. injection of 1.5 mg/kg body weight Rocuronium (Esmeron®; EssexPharma, Munich, Germany) allowed single-breath-stop micro-CT within 40 s scan time (as described below). Relaxation afterwards was reversed by i.p. injection of 20 mg/kg body weight of Sugammadex (Bridion®; EssexPharma).

For micro-CT imaging the mice were fixed in a custom-made acrylic cradle that was mounted onto the three-jaw drill chuck of the rotational axis of the micro-CT. An industrial x-ray inspection system (Yxlon Y.Fox; Yxlon International GmbH, Hamburg, Germany) was used. The system was equipped with a multifocus cone beam x-ray source with a diamond-coated high-power tungsten target and a 12-bit direct digital flat bed detector (Varian PaxScan 2520; Varian, Palo Alto, CA, USA). The tube parameters were set to 80 kV and 75 µA (focal spot size 5 µm). The scanning protocol was programmed to acquire images at 30 frames per second (fps) while continuously rotating the mouse by 190° (180° plus 10° cone beam angle) within 40 seconds scan time, resulting in a total of 1200 projections per scan [20]. The projections were reconstructed using a filtered backprojection algorithm with a matrix of 512×512×512 using the software provided by the manufacturer of the micro-CT (Reconstruction Studio; Yxlon International GmbH).

### Characterization of the time course of contrast enhancement

In the first part of the study, the time course of the uptake of two formulations of 100 µl of ExiTron nano (ExiTron nano 6000 and ExiTron nano 12000; n = 3 for each group) by the RES of the liver and spleen was assessed in healthy C57BL/6J mice.

Since our micro-CT does not provide Hounsfield units, we measured the relative increase of the contrast enhancement compared to a baseline level. More exactly, the density of the liver and vessels in non-enhanced images was set to 100%. After injection of the contrast agent repetitive micro-CT scans were performed at different time points (as described in the results section) for up to 6 months. The distribution pattern and the relative density (in % as compared to the non-enhanced baseline levels) of the contrast agent within the vascular system (ROI measurements performed within the left ventricle) and the liver (ROI measurements performed avoiding large intrahepatic vessels) were analysed. During this period mice were weighed and checked for their well-being every other day.

### Mouse model of liver metastasis

To test the feasibility of the detection of liver metastases using the nanoparticular contrast agent, intrasplenic injection of MC38 or C15-A.3 colon carcinoma cells was performed in anesthetized C57BL/6 Han TgN (CEAgen) HvdP mice expressing the human Carcinoembryonic antigen (CEA) as a transgene. MC38 is a sygeneic Methyl-Cholanthren-induced colon cancer line, while C15-A.3 is a MC38-derivative cell line transfected with the CEACAM5 gene coding for the human Carcinoembryonic antigen (CEA). In some of the mice receiving MC38 cells a splenectomy was performed 7 days after tumor cell injection in order to prevent rupture of the spleen due to the rapid primary tumor growth before the development of liver metastases.

Since the time until development of liver metastases varied, repetitive scans were performed to detect liver metastases and to monitor tumor growth. For this purpose, micro-CT examinations were performed after a single intravenous injection of 100 µl ExiTron nano 6000 (15 mice) or 100 µl ExiTron nano 12000 (15 mice) prior to the first micro-CT scan, which was performed on day 9 after splenic tumor engraftment.

### Image analysis

Analysis of reconstructed images was performed using the public domain software OsiriX (v3.5.1; www.osirix-viewer.com).

For characterization of the time course of liver uptake and intravascular contrast, ROIs (regions of interest) were placed in coronal slices of the liver and the left ventricle. While positioning ROIs in the liver, care was taken to avoid large liver vessels, as this could artificially influence liver tissue values. The values were averaged (mean±1SD) to achieve the vessel and liver CT contrast enhancement curves for both ExiTron nano 6000 and ExiTron nano 12000. In mice developing liver metastases the diameter of the liver lesions was measured using OsiriX software and the smallest identifiable liver metastasis was determined.

## Results

### Time course characterization of contrast enhancement

All mice tolerated the injection of both formulations of ExiTron nano well and even after 6 months post injection of the contrast agent were no adverse effects, such as weight loss or abnormal behaviour, observed.

**Figure 1:**
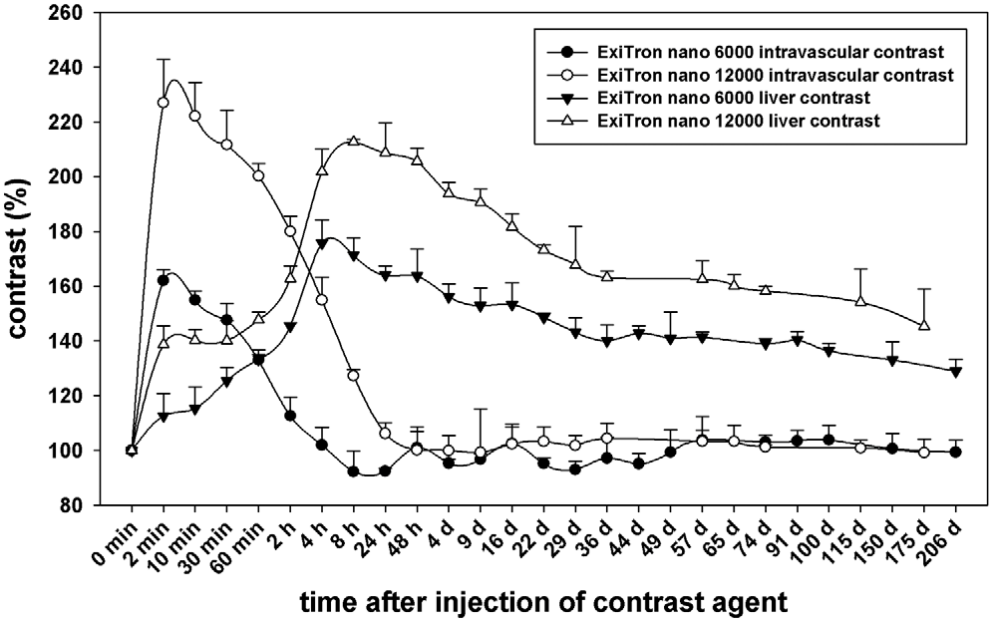
Time course of contrast enhancement within the vascular system and the liver of C57BL/6J mice (n = 3 per group) after a single i.v. injection of 100 µl ExiTron nano 6000 or ExiTron nano 12000. Measurements were performed by placing a ROI within the left ventricle (vessel contrast) and within the liver avoiding large intrahepatic vessels. The baseline level ( = 100%) refers to measurement of the relative density of the liver and the vascular system prior to administration of contrast agent.

**Figure 2:**
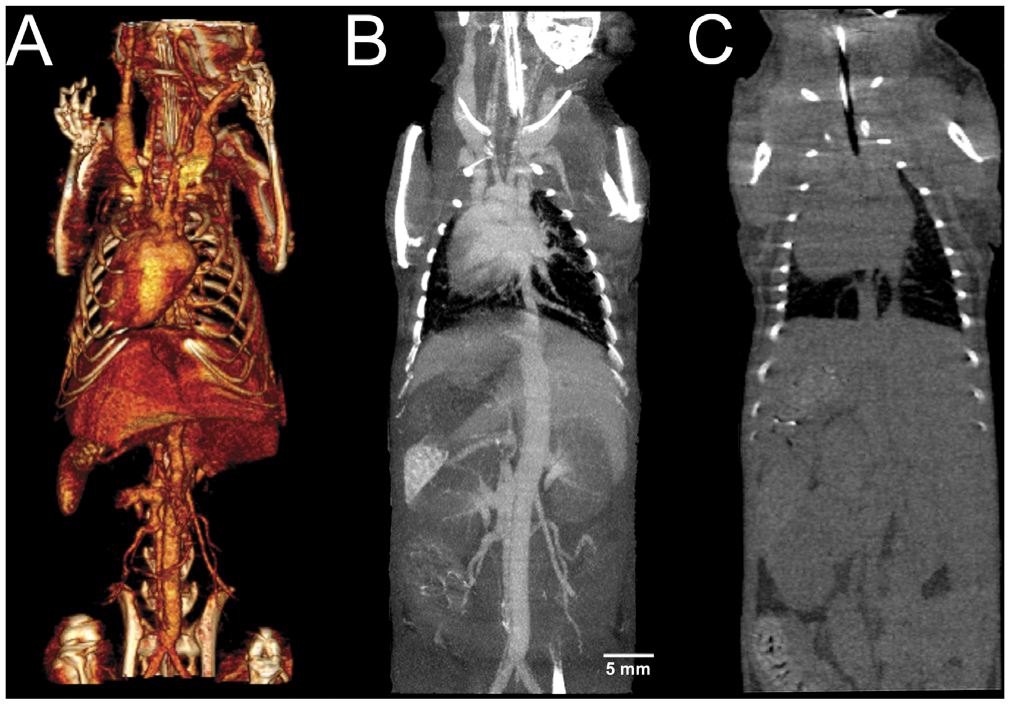
A shows a volume rendering of a mouse 30 minutes after i.v. injection of ExiTron nano 12000. B is a curved maximum intensity projection in coronal orientation of the same scan. A and B demonstrate the feasibility to perform CT angiography during the early intravascular phase of the tested contrast agent. Additionally, A and B show the early contrast agent uptake by the RES with increasing contrast of liver and spleen. C is a coronally oriented curved maximum intensity projection of a mouse that did not receive contrast agent.

Two minutes post injection of ExiTron nano 6000 and 12000 the vessel density peaked, and subsequently halved after 60 minutes or 4 hours, respectively (Fig. 1). At both concentrations, vessels were clearly visible. ExiTron nano 12000, however, provided the stronger contrast (Fig. 2), and a more prolonged time window for vessel imaging of up to 4–8 hours after contrast agent injection. Vessel contrast returned to baseline values 4 or 24 hours after injection of ExiTron nano 6000 or 12000, respectively.

While the intravascular contrast decreased, liver contrast increased and reached its peak 4 h after injection of ExiTron nano 6000 and 8 h after injection of ExiTron nano 12000. The initially observed rapid increase (small plateau) of liver contrast observed 2–10 minutes after injection of ExiTron nano 6000 and 12000 is not only attributable to early uptake by the RES of the liver but also to the presence of contrast agent located within the vascular system of the liver. Liver imaging was feasible even after more than 200 days post administration of a single injection of ExiTron nano 6000 or 12000.

**Figure 3:**
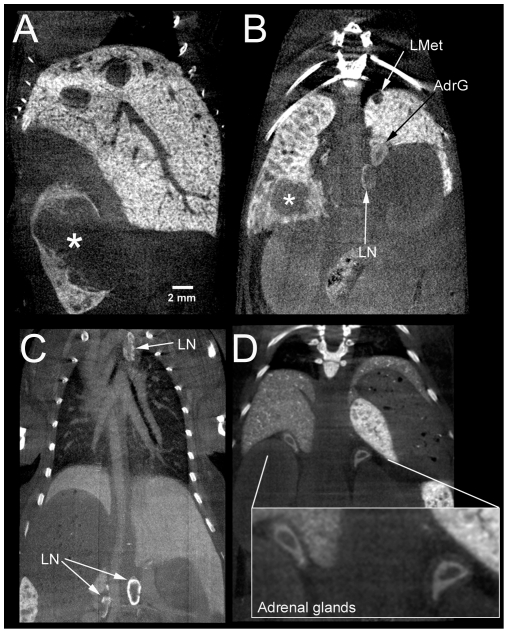
A and B show intrasplenic (*) and intrahepatic (LMet) growing tumors 26 days after intrasplenic injection of C15A3 colon tumor cells. A and B were acquired 4 hours after i.v. injection of 100 µl ExiTron nano 12000. B, C, and D illustrate contrast enhancement of the abdominal and mediastinal lymph nodes (LN) and of the adrenal glands (AdrG). C was acquired 4 hours after i.v. injection of 100 µl ExiTron nano 12000; D was acquired 22 days after i.v. injection of 100 µl ExiTron nano 12000. Micro-CT scanning parameters: 40 sec scan time; 190° rotation; 1200 projections; voxel size 41×41×55 µm^3^.

**Figure 4:**
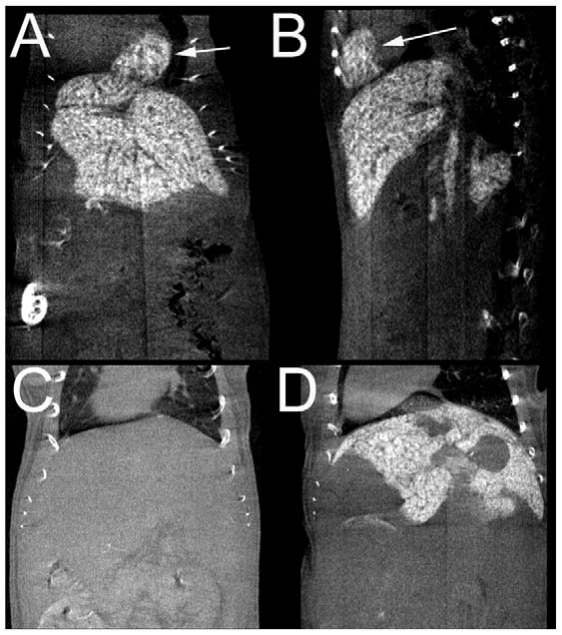
A and B show a partial diaphragmatic herniation of the left upper liver lobe in coronally (A) and sagittally (B) reconstructed maximum intensity projections of a C57BL/6J mouse 22 hours after i.v. injection of 100 µl ExiTron nano 12000. The herniated liver tissue can be easily delimited from the adjacent heart due to the positive liver contrast. C and D are micro-CT scans of the murine liver before (C) and 24 hours after (D) intravenous administration of 100 µl ExiTron nano 6000.

In addition to contrast enhancement of liver and spleen, we most interestingly observed contrast enhancement of the abdominal and mediastinal lymph nodes, mainly located in the marginal area of the nodes after injection of both formulations of ExiTron nano (Fig. 3C). We furthermore also identified evident contrast enhancement of the adrenal glands, mainly located in the cortex and here predominantly within the zona reticularis (Fig. 3D). As an incidental finding in one mouse we observed diaphragmatic herniation of a part of the left medial liver lobe, which was distinctively delimited from the adjacent heart tissue due to use of the liver-specific contrast agent (Fig. 4A and 4B). Figures 4C and 4D were inlcuded to provide a comparison of the liver in an animal before (Fig. 4C) and 24h after i.v. administration of 100 µl ExiTron nano 6000 (Fig. 4D).

### Monitoring development of liver metastasis by repetitive micro-CT

**Figure 5:**
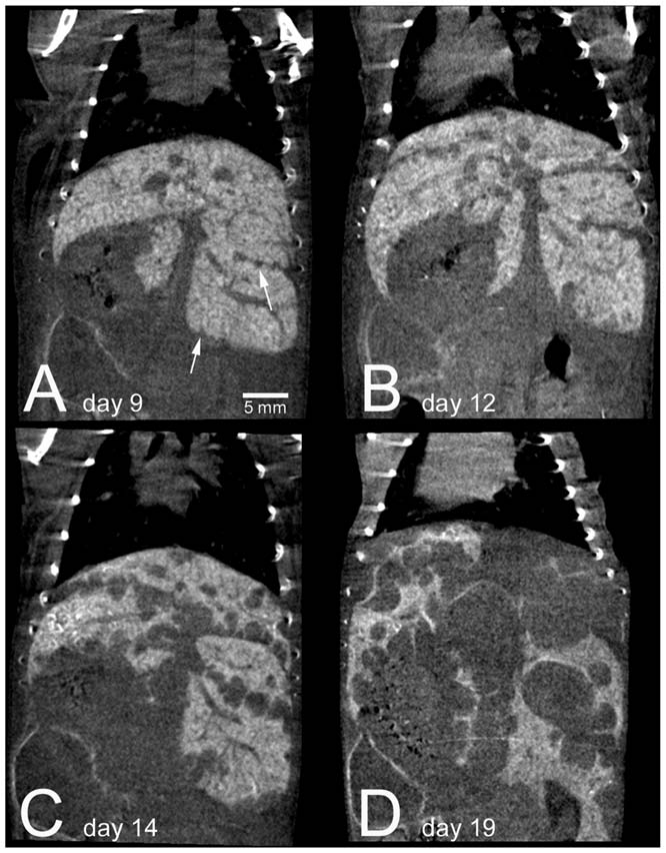
Repeated micro-CT of a mouse illustrates development of liver metastases 9, 12, 14, and 19 days after intrasplenic injection of MC38 colon tumor cells. Smallest detectable liver metastases (arrows) measured 300 µm in diameter.

Longitudinal monitoring of liver lesions was feasible after a single injection of ExiTron nano 6000 (Fig. 5A-D). Liver metastases did not take up the contrast agent, making them clearly detectable as unenhanced regions within the hyperdense healthy liver tissue. The smallest detectable liver metastases measured approx. 300 µm in diameter (Fig. 5A). The strong contrast enhancement additionally allowed delineation of intrasplenic tumors (* in 3A and 3B). As reported in the methods section, some mice underwent splenectomy due to massive intrasplenic tumor growth in order to allow further monitoring of liver metastases: in these animals we observed liver uptake of ExiTron nano to be increased by approx. 15% (p>0.05).

## Discussion

Micro-CT imaging of small liver lesions in mice relies on the use of contrast agents to compensate for the insufficient soft tissue contrast in non-enhanced liver tissue. While contrast agents are normally used to contrast the pathological process itself, in liver imaging contrast agents are frequently used to increase the contrast of cells located within the healthy liver tissue, hereby visualizing pathological processes as unenhanced regions within the contrasted liver tissue [4], [5], [6], [7], [8], [13], [14], [21]. To identify very small liver lesions, a contrast agent needs to provide a possibly strong and specific contrast of the liver tissue. Since in animal experiments longitudinal measurements of pathologic lesions are frequently performed to investigate pathological processes and to monitor the efficacy of all kinds of therapeutic regimens, it would be ideal if a long-lasting contrast could be obtained after a single injection of the contrast agent, hereby eliminating the need for time-consuming and cumbersome repeated tail vein injections.

In this study we present the first results using two novel alkaline earth metal-based nanoparticulate contrast agents (ExiTron nano 6000 and 12000) for preclinical computed tomography, which were found to provide a strong contrast of the liver lasting up to 6 months. The tested contrast agents differ in several aspects from other commercially available contrast agents. Firstly, contrast of the liver and spleen were achieved by uptake of the nanoparticles by the RES, while other most frequently used contrast agents are selectively taken up by hepatocytes via an apolipoprotein E (ApoE) receptor-mediated pathway [6], [8], [12]. Most interestingly, we found that besides liver and spleen uptake, the agent also provided strong contrast of the abdominal and mediastinal lymph nodes as well as of the adrenal glands, mainly in the zona reticularis between the cortex and the medulla. While liver and splenic uptake can be explained by RES-mediated uptake of nanoparticles, the reason for the observed contrast enhancement in the periphery of the lymph nodes (Fig. 3C) and in the adrenal glands (Fig. 3D) can be expected to be due to macrophages, as discussed by Weinmann et al [22].

A similar contrast agent biodistribution has, to the authors' best knowledge, not been described before in mouse studies. We have, however, experienced that other contrast agents, e.g. Fenestra LC, also result in a relatively weak and infrequent enhancement of the lymph nodes and adrenal glands (unpublished data of our workgroup). Likewise, Martiniova et al. performed micro-CT after injection of Fenestra LC in a model of metastatic pheochromocytoma [13], and reported that adrenal gland lesions were usually undetectable on micro-CT unless they were large and growing into the contrast-enhanced liver area, corroborating our observations.

The second difference compared to other liver contrast agents is the smaller injection volume required. Especially in mice, which have a total blood volume in the range of 1200–1500 µl, the injection of larger volumes of a contrast agent can be problematic. Thus, an injection volume of 100 µl is much more preferable over injection volumes between 200 µl and up to 1500 µl, which are usually recommended for iodine-based contrast agents [3], [4], [6], [7], [8], [14], [15], [23], [24], [25] to provide sufficient contrast of the liver. The reduced injection volume is facilitated by the high concentration of the alkaline earth metal, which is possible due to the contrast agents' nanoparticulate formulation.

Due to the strong contrast of ExiTron nano and the slightly delayed uptake by the RES, not only imaging of the liver and spleen but also visualization of the vasculature was feasible directly after injection of the contrast agent and up to 30 minutes (ExiTron nano 6000) or 240 minutes (ExiTron nano 12000) post injection. These time windows are similar to those of other frequently used blood pool contrast agents [23]; however, the latter require unfavorable higher injection volumes to obtain similar contrast. Though vessel imaging was feasible with both ExiTron nano 6000 and ExiTron nano 12000, ExiTron nano 12000 was preferable due to its higher contrast and increased time window.

Imaging of the liver was possible from about 30 minutes after contrast agent injection, with the contrast peaking after 4–8 hours and lasting up to several weeks and months, which greatly differs to other contrast agents where liver contrast values return to baseline levels within a few days after the injection [3], [5], [6], [9], [10], [11], [21], [26]. Though significant liver contrast was reported as persisting for example, for 1 week [3] and up to 15 days [6] after a single injection of Fenestra LC, image quality at these late time points was reduced. In our study the smallest detectable liver metastasis measured approx. 300 µm in diameter.

To conclude, the nanoparticulate contrast agents ExiTron nano 6000 and 12000 provide rapid, strong and specific contrast of the RES of the liver and the spleen at low injection volumes. Due to the long-lasting contrast of the liver up to several weeks after injection, ExiTron nano allows longitudinal imaging of e.g. developing liver metastasis after a single injection of the contrast agent, which saves time and reduces additional stress for the animals. The high intravascular contrast up to 4–8 hours after injection of the contrast agent furthermore allows this contrast agent to be employed in the field of vascular imaging. Finally, strong enhancement of lymph nodes and adrenal glands renders this contrast agent an interesting tool for future studies of these organs in small animal models.
